# Evaluation of tumor-colonizing *Salmonella* strains using the chick chorioallantoic membrane model

**DOI:** 10.1128/mbio.03590-24

**Published:** 2025-01-28

**Authors:** Khin K. Z. Mon, Linda J. Kenney

**Affiliations:** 1 Department of Biochemistry and Molecular Biology, University of Texas Medical Branch, Galveston, Texas, USA; 2 Sealy Center for Structural Biology, University of Texas Medical Branch, Galveston, Texas, USA; National University of Singapore, Singapore, Singapore

**Keywords:** chick chorioallantoic membrane, tumor-colonizing *Salmonella*, CAM tumor, therapeutic agent, 3D tumor spheroids

## Abstract

The chick embryo chorioallantoic membrane (CAM) tumor model is a valuable preclinical model for studying the tumor-colonizing process of *Salmonella enterica* serovar Typhimurium. It offers advantages such as cost-effectiveness, rapid turnaround, reduced engraftment issues, and ease of observation. In this study, we explored and validated the applicability of the partially immune-deficient CAM tumor model. Herein, we demonstrate that *Salmonella* preferentially colonizes tumors and directly causes tumor cell death. Bacterial migration, tumor colonization, and intra-tumor distribution did not require flagellar-mediated motility. The vast majority of *Salmonella* that colonized the CAM tumor were extracellular. Thus, tumor invasion was independent of both *Salmonella* pathogenicity island-1-encoded and *Salmonella* pathogenicity island-2-encoded type III secretion systems. Surprisingly, the extracellular residence of *Salmonella* on CAM tumors did not require biofilm formation. We evaluated our wild-type parental strain compared to the attenuated clinical strain VNP20009 and discovered a reduced tumor colonization capability of VNP20009. The inability to effectively colonize CAM tumors potentially explains the reduced anti-tumor efficacy of VNP20009. Our work establishes the xenograft CAM model as an informative and predictive screening platform for studying tumor-colonizing *Salmonella*.

**IMPORTANCE:**

Cancer has a major impact on society, as it poses a significant health burden to human populations worldwide. *Salmonella* Typhimurium has demonstrated promise in cancer treatment by exerting direct tumoricidal effects and enhancing host-mediated anti-tumor immunity in xenograft mouse studies. A general understanding of its pathogenesis and the relative ease of genetic manipulation support the development of attenuated strains for therapeutic use. Alternative *in ovo* models, such as the chorioallantoic membrane tumor model, present a suitable screening platform to accelerate the development of therapeutic strains. It allows for rapid evaluation of *Salmonella* strains to assess their efficacy and potential as oncolytic agents. The present study establishes that the *in ovo* tumor model can be utilized as a preclinical tool for evaluating oncolytic *Salmonella*, bridging the gap between *in vitro* and *in vivo* screening.

## INTRODUCTION

In the field of bacterial-mediated cancer therapy, *Salmonella enterica* serovar Typhimurium (STm) has emerged as an ideal therapeutic candidate. This is in part due to its intrinsic tumor targeting ability (1), its facultative anaerobic properties, which allow colonization in both hypoxic and oxygenated regions of heterogeneous tumor microenvironments (2), its invasive capability (3), and its direct or immune-mediated anti-tumor effects (3–6). STm infection mainly causes self-limiting gastrointestinal disease in healthy humans but can sometimes manifest as extraintestinal systemic infections in immune-compromised individuals (7). To promote its safe application in cancer patients, bacterial pathogenic properties need to be eliminated or attenuated without compromising the tumor-colonizing capability, and thus subsequent anti-tumor potency. Therefore, tuning the balance between virulence attenuation and tumor-colonization capability is an important criterion in the design of suitable STm strains.

The chick embryo chorioallantoic membrane (CAM) has long been used as a robust model for cancer and cardiovascular research, as well as for preclinical evaluation of cancer drug therapies (8–10). The CAM model is an immunodeficient model that is receptive to xenografting of various mammalian tumor cells without inducing a host immune response (11). The well-vascularized embryonic tissue of the CAM supports and preserves heterogeneous tumor growth with a much shorter experimental timeline (typically 3–4 days) for tumor development compared to mouse models, which requires weeks (12). Moreover, the CAM model also allows real-time visualization of tumor growth (13) as well as the dynamics of bacterial infection via fluorescence stereomicroscopy (14). These attributes indicate that the CAM tumor model could be well suited for preliminary evaluation of *Salmonella*-based therapies for cancer treatment.

The engineered STm strain VNP20009 is attenuated by purine auxotrophy (a deletion in *purI*) and lipid A modification (a deletion in *msbB*) (15). It demonstrated an excellent safety profile with observable tumor growth retardation in a variety of preclinical xenograft mouse models (16–19). Despite the high success rate in the mouse studies, no anti-tumor effect was observed in humans. The intravenous infusion of the VNP20009 strain to melanoma patients in a clinical trial failed and was attributed to low bacterial tumor colonization and rapid bacterial clearance by the host immune response (20). The failure of the VNP20009 strain to colonize tumors highlighted the xenograft mouse model as a poor predictor of therapeutic success in humans. Hence, there is a need to explore alternative models to overcome some of the translational limitations associated with traditional mouse models. We hypothesized that the CAM tumor model might serve as a preliminary screening platform to study the tumor-bacterial interactions to evaluate the efficacy of tumor-targeted *Salmonella* strains for therapeutic purposes.

In the current work, we developed the *in ovo* CAM tumor model and compared it with an *in vitro* three-dimensional (3D) tumor spheroid model to examine the relevance of the screening data obtained (21). While 3D tumor spheroids can mimic *in vivo* solid tumor features such as structural organization, cellular assembly, and nutrient gradients (22), they cannot fully simulate the heterogeneous tumor microenvironment established within a living host. Therefore, the CAM tumor model serves as a better preclinical model, bridging an informational gap between *in vitro* cell-based models and *in vivo* animal-based systems for screening oncolytic *Salmonella*. With the CAM tumor model, we demonstrated that STm pathogenic traits such as flagella, virulence factors, and biofilm formation were not essential for tumor colonization. We further discovered that reduced tumor colonization by VNP20009 was attributed to its growth sensitivity within the tumor microenvironment, which subsequently impacted its anti-tumor effectiveness.

## RESULTS

### Validation of the CAM model for studying tumor-colonizing *Salmonella*

**Fig 1:**
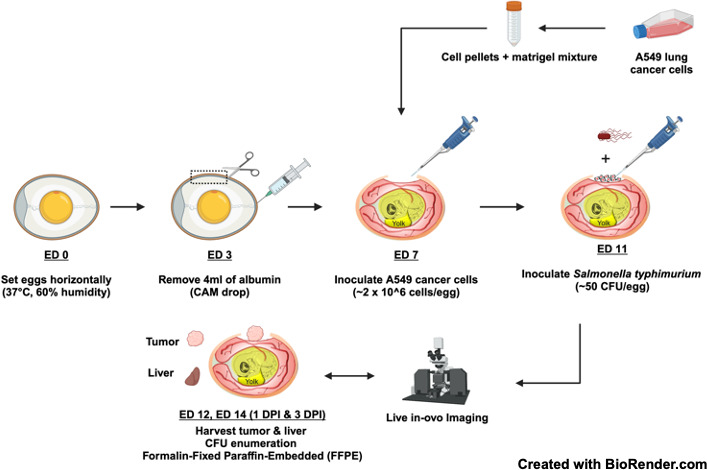
Experimental scheme of the CAM tumor STm infection model.

A general overview of the CAM tumor STm infection model is depicted in Fig. 1. We first determined the infectious dose of STm by infecting tumor-bearing chick embryos with different doses of bacteria: approximately 500 colony-forming units (CFU), 50 CFU, or 5 CFU. Chick embryo mortality was monitored daily post-bacterial infection with phosphate-buffered saline (PBS) as the control group. The highest inoculum of ~500 CFU caused the greatest mortality (38% survival) at 1 day post-infection (DPI) compared to groups that received lower dosages of ~50 CFU (66% survival) or ~5 CFU (77% survival) (Fig. S1). To minimize the mortality of highly susceptible tumor-bearing chick embryos as well as to ensure effective tumor colonization, we selected the mid-range of ~50 CFU as the optimal infectious dose.

**Fig 2:**
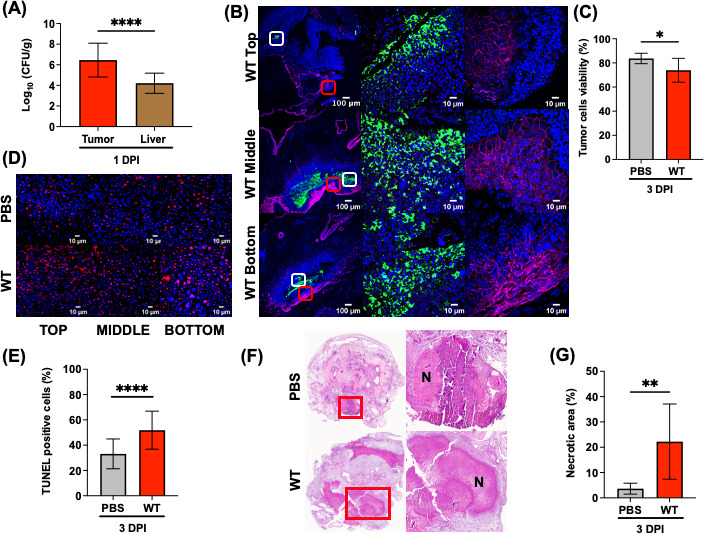
STm promotes tumor cell death in the CAM model. (**A–G**) Chick embryos with pre-established A549 tumors were directly inoculated with *Salmonella* WT or PBS on ED11. Tumors and livers were harvested at 1 and 3 DPI. (**A**) *Salmonella* WT CFU recovered per gram of tissue from tumors and livers at 1 DPI (tumor to liver ratio 1,000:1). (**B**) Representative images of *Salmonella* WT LPS (green), E-cadherin (magenta), and Hoechst (blue) staining detected in tumor sections from top, middle, and bottom of whole tumors harvested at 1 DPI. Scale bar = 100 µm (10× objective) and 10 µm (100× objective). The middle panel is the zoomed image of the white box and the right panel is the zoomed image of the red box at 100× objective. (**C**) A trypan blue exclusion assay was performed on *ex vivo* dissociated tumor cell suspensions, indicating a loss of tumor cell viability in the group infected with WT *Salmonella* at 3 DPI. (**D**) Representative images of terminal deoxynucleotidyl transferase-mediated deoxyuridine triphosphate nick end-labeling (TUNEL)-stained (red) and Hoechst (blue) tumor sections from 3 DPI (top, middle, and bottom). Scale bar = 10 µm, 100× objective. (**E**) Quantitative comparison of the percentage of TUNEL-positive nuclei between WT-infected tumors (52%) and PBS-treated tumors (33%) at 3 DPI. (**F**) Representative images of hematoxylin and eosin (H&E) tumor sections. Zoomed images of the red box of the necrotic region at 3 DPI. N: necrotic areas. (**G**) The percentage of the necrotic area as quantified by an external pathologist from H&E-stained tumor sections infected with WT (22%) and PBS (4%) at 3 DPI. The results represent the mean ± standard deviation (SD). For all, the statistical significance was determined by unpaired *t*-test, not significant (ns). *P ≤ 0.05, **P ≤ 0.01, ***P ≤ 0.001, and ****P ≤ 0.0001.

The important features of tumor-targeting STm are the higher tumor specificity in colonization compared to organ colonization and the ability to penetrate tumor tissue (23, 24). These criteria were tested first in the CAM tumor model. At 1 DPI, wild-type (WT) STm accumulated preferentially in tumors over livers of chick embryos at a ratio of 1,000:1. The mean CFU/g in tumors was 1.9 × 10^8^ CFU/g compared to 1.9 × 10^5^ CFU/g in livers (Fig. 2A, note log scale). Next, we examined the intra-tumoral distribution of STm. Infected whole tumors were harvested, embedded in paraffin, and sectioned at different tissue depths (top, middle, and bottom). The tumor sections were stained with antibodies against bacterial lipopolysaccharide (green), nuclei (blue), and E-cadherin (magenta) and examined by confocal microscopy. STm was observed at all tumor tissue depths as evident by lipopolysaccharide staining (LPS = green, second panel in Fig. 2B and Fig. S2). Specifically, bacteria were mostly present in the metastatic tumor cell regions, where decreased E-cadherin staining indicated a loss of cell-to-cell adhesion junctions.

### STm colonization drove direct tumor apoptosis in the CAM model

We next tested the hypothesis that STm infection directly promotes tumor cell death. A trypan blue exclusion assay was performed on dissociated tumor cells to test *ex vivo* tumor cell viability. At 3 DPI, STm infection significantly reduced the percentage of tumor cell viability (74%) compared to PBS-treated tumors (84%) (Fig. 2C). Because the presence of STm was detected throughout the entire tumor (Fig. 2B), it raised the question of whether its localization directly induced tumor cell apoptosis. We utilized the terminal deoxynucleotidyl transferase-mediated deoxyuridine triphosphate nick end-labeling (TUNEL) assay to detect *Salmonella*-induced DNA fragmentation in tumor cells. A marked accumulation of TUNEL-positive cells (red signals) was observed in STm-infected tumors relative to PBS-treated tumor sections at the top, middle, and bottom levels (Fig. 2D). Immunofluorescence images of TUNEL-stained tumor sections were further quantified using Fiji software (25). The percentage of TUNEL-positive nuclei in STm-infected tumor tissue sections was significantly higher than in PBS tumors (52% vs 33%, respectively), indicating that tumor cells underwent increased apoptosis following bacterial infection (Fig. 2E). Lastly, the histological changes in hematoxylin and eosin (H&E)-stained tumor sections were analyzed. A more extensive necrotic area in *Salmonella*-infected tumors (22%) was detected compared to the PBS control group (4%) (Fig. 2F and G).

### Bacterial motility was not required for tumor colonization or intra-tumoral distribution

**Fig 3:**
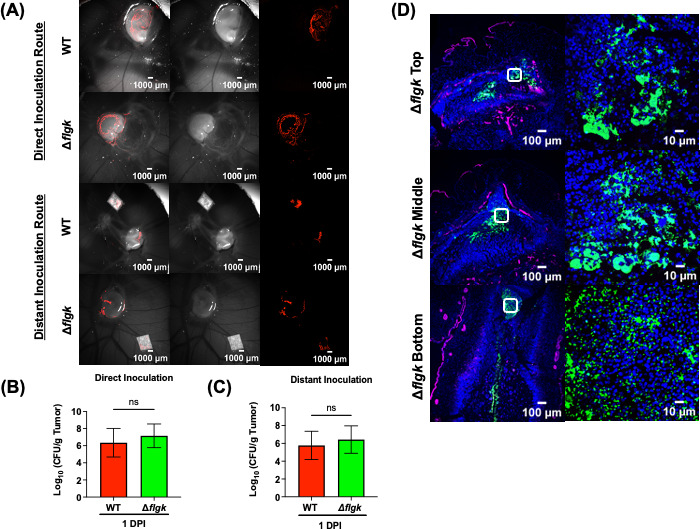
Flagellar motility is not required for tumor colonization or intra-tumoral distribution. (**A**) Representative live *in ovo* images of mCherry-tagged *Salmonella*. WT and *ΔflgK* colonizing A549 tumors on CAMs via either direct (bacteria inoculated directly on tumors) or distant inoculation (bacteria deposited on filter paper, ~0.5 cm away from tumors) at 1 DPI. (**B and C**) Total *Salmonella* WT and *ΔflgK* CFU recovered per gram of tumor tissue at 1 DPI from direct and distant inoculation. The results represent the mean ± SD. The statistical significance was determined by unpaired *t*-test, not significant (ns). (**D**) Representative images of *Salmonella ΔflgK* LPS (green), E-cadherin (magenta), and Hoechst (blue) staining detected in tumor sections from top, middle, and bottom levels of whole tumors harvested at 1 DPI. Scale bar = 100 µm (10× objective) and 10 µm (100× objective). The second column is the zoomed in details of the white box at 100× objective.

The prevailing hypothesis regarding a role for flagella in tumor colonization is that motility is essential for bacteria to effectively target, colonize, and disperse within the tumor environment (26–28). To test this hypothesis, WT or a *ΔflgK* null strain was directly inoculated onto CAM tumors. The *flgK* gene encodes a flagellar hook-associated protein; its deletion renders *Salmonella* non-motile (29). Live *in ovo* images of the bacterial colonization pattern, as well as CFU enumeration from tumors, were comparable between the WT and the *ΔflgK* null strain (Fig. 3A and B). We reasoned that the role of flagella might be dispensable when bacteria were directly inoculated onto the tumor, rendering colonization a passive process. Therefore, we also tested a distant inoculation route by depositing bacteria a short distance away from the tumor (~0.5 cm) on filter papers to test whether *Salmonella* required flagellar-mediated motility for tumor colonization. The *ΔflgK* strain showed no defect in CAM tumor colonization and colonized tumors to the same extent as the WT (Fig. 3A and C). The intra-tumoral distribution pattern of the non-motile *ΔflgK* strain was also comparable to that of the motile WT (Fig. 2B), indicating that the *ΔflgK* strain was able to disperse and penetrate all depths of the tumor (Fig. 3D). These results provided strong evidence that STm migration, tumor colonization, and intra-tumoral distribution were not dependent on bacterial motility in the CAM tumor model.

### 3D tumor spheroids do not recapitulate the CAM tumor model with respect to key virulence factors encoded on pathogenicity islands 1 and 2

**Fig 4:**
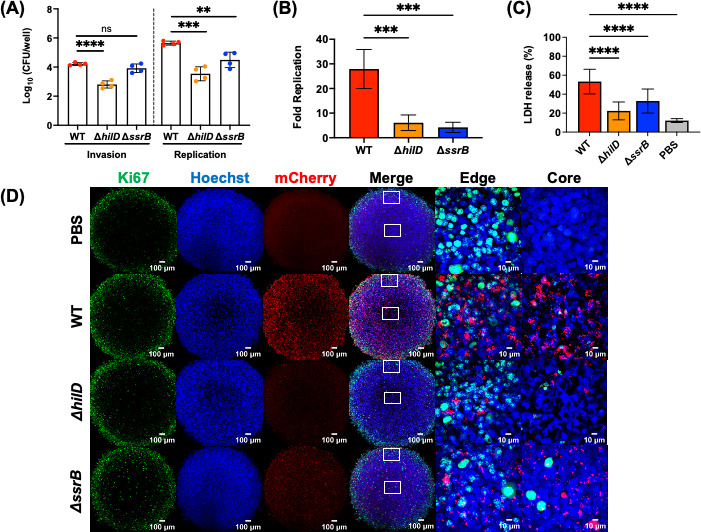
SPI-1 and SPI-2 are required for intracellular replication of STm within 3D tumor spheroids. (**A**) Intracellular bacterial enumeration of STm strains (WT, *ΔhilD*, and *ΔssrB*) in A549 tumor spheroids measured at invasion (mean log CFU/well at 2 HPI: WT = 4.2, *ΔhilD* = 2.8, *ΔssrB* = 3.9) and replication (mean log CFU/well at 16 HPI: WT = 5.6, *ΔhilD* = 3.5, *ΔssrB* = 4.5) stages of infection. Dots: biological replicates, each with a minimum of five technical replicates. The bars represent the mean ± SD. (**B**) Fold replication (16 HPI/2 HPI) of each STm strain (WT = 28-fold, *ΔhilD* = sixfold, *ΔssrB* = fourfold) within A549 tumor spheroids. The bars represent the means of four biological replicates, a minimum of five technical replicates each. Error bars ± SD. (**C**) The amount of LDH released from A549 tumor spheroids after infection with STm (WT = 53% , *ΔhilD* = 22%, and *ΔssrB* = 33%) at 16 HPI. The bars represent the means of three biological replicates, a minimum of five technical replicates each. Error bars ± SD. For all, the statistical significance was determined by one way analysis of variance (ANOVA) with Sidak’s multiple comparison test. Not significant (ns), *P ≤ 0.05, **P ≤ 0.01, ***P ≤ 0.001, and ****P ≤ 0.0001. (**D**) Representative images of immunofluorescence-labeling of 3D tumor spheroids with Ki67 AlexaFluor 488 (green), Hoechst (blue), and mCherry-tagged bacterial strains (red) at both 10× and 100× objectives (edge and core of spheroids). Maximum intensity projections of Z-stack images were taken with an Olympus Super Resolution Spinning Disk SpinSR-10 microscope. Scale bar = 100 µm (10×) and 10 µm (100×).

In the host gastrointestinal tract, STm utilizes a type III secretion system (T3SS1) encoded by *Salmonella* pathogenicity island-1 (SPI-1) to invade host intestinal epithelial cells and an SPI-2-encoded T3SS2 for intracellular growth and survival within host macrophages (30–33). We, therefore, investigated whether bacterial invasion or replication within tumors was also dependent on SPI-1 and SPI-2. To address this question, we first employed the *in vitro* 3D tumor spheroid model before assessing their roles *in vivo* in the CAM tumor model. The use of 3D magnetic cell culture technology produced structurally relevant tumor spheroids that mimicked some features of *in vivo* solid tumors. The spatial architecture with regard to oxygen and nutrient gradients, extracellular matrix, and cell-to-cell interactions is maintained (21, 22). Within tumor spheroids, intracellular invasion and replication rates of STm strains were examined using a gentamicin protection assay. The elimination of *hilD* (the SPI-1 master regulator) resulted in a significant invasion defect with a mean reduction of 1.4 log CFU/well compared to WT (Fig. 4A), suggesting that SPI-1 played a significant role in early bacterial invasion of tumor cells. At 16 hours post-infection (HPI), an intracellular replication rate of both Δ*hilD* (mean log CFU/well = 3.5) and the SPI-2 master regulator, Δ*ssrB* (mean log CFU/well = 4.5) were significantly reduced relative to WT (mean log CFU/well = 5.6) (Fig. 4A). Consequently, the overall replication rate (16 HPI/2 HPI) was higher for WT (28-fold) compared to either Δ*hilD* and Δ*ssrB* strains (sixfold and fourfold, respectively) (Fig. 4B). We next examined the cytotoxic damage induced by bacterial infection of 3D tumor spheroids using a lactate dehydrogenase (LDH) assay 16 HPI. The amount of LDH release is directly correlated with cell cytotoxicity levels. Tumor spheroids infected with WT bacteria released higher amounts of LDH (53% of total maximum LDH release at 100%) compared to SPI-1-deficient (22%) or SPI-2-deficient (33%) strains (Fig. 4C). Whole-mount immunofluorescence staining of 3D tumor spheroids exhibited a specific cellular structural organization. Proliferating tumor cells were observed in the periphery, with an inner layer of quiescent cells and a necrotic core region. These were evident by distinct fluorescence intensity profiles of the cell proliferation marker Ki67, in which the intensity varied from high to low from the periphery to the core (Fig. 4D). From representative images of infected 3D tumor spheroids at 16 HPI, both SPI-1-deficient and SPI-2-deficient strains were observed at reduced levels compared to the WT (Fig. 4D) consistent with the quantification of CFU (Fig. 4A).

**Fig 5:**
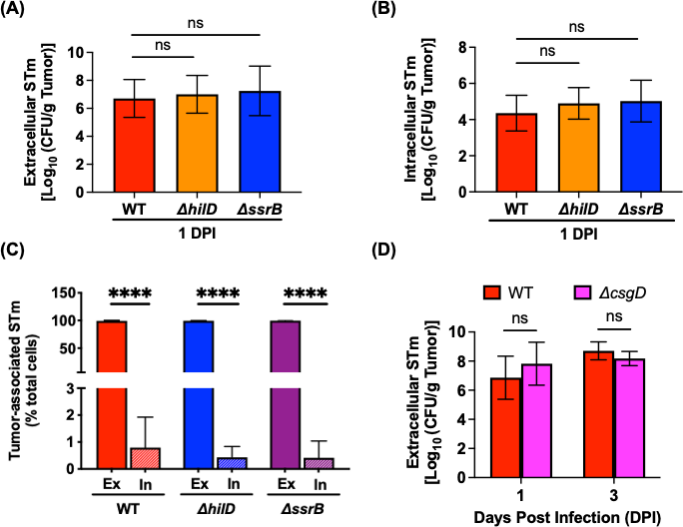
In the CAM tumor model, STm resides in an extracellular, non-biofilm state. (**A**) Extracellular CFU recovered after infection with WT *Salmonella*, *ΔhilD*, and *ΔssrB* per gram of tumor at 1 DPI. Statistical significance was determined by one-way ANOVA with Sidak’s multiple comparison test. (**B**) Intracellular WT *Salmonella*, *ΔhilD*, and *ΔssrB* CFU recovered per gram of tumor at 1 DPI. Statistical significance was determined by one-way ANOVA with Sidak’s multiple comparison test. (**C**) Percentage of total tumor-associated STm (WT, *ΔhilD*, and *ΔssrB*) recovered before and after an *ex vivo* gentamicin assay from infected CAM tumors at 1 DPI. EX, extracellular; IN, intracellular. Statistical significance was determined by one-way ANOVA with Sidak’s multiple comparison test. (**D**) Extracellular *Salmonella* WT and *ΔcsgD* CFU recovered per gram of tumor at 1 and 3 DPI. The results represent the mean ± SD. The statistical significance was determined by unpaired *t*-test. For all, not significant (ns), *P ≤ 0.05, **P ≤ 0.01, ***P ≤ 0.001, and ****P ≤ 0.0001.

We then investigated whether tumor colonization and intracellular invasion or replication required SPI-1 or SPI-2 in the CAM tumor model. Using an *ex vivo* gentamicin assay, we quantified total tumor-associated bacteria (i.e., both extracellular and intracellular bacteria) recovered from the CAM tumors. Both SPI-1 (*ΔhilD*)-deficient and SPI-2 (*ΔssrB*)-deficient strains were found to colonize (extracellular) and invade (intracellular) tumor cells to the same extent as WT at 1 DPI (Fig. 5A and B). In the mouse tumor model, *Salmonella* is found almost exclusively extracellularly, forming biofilms on tumors (34). We, therefore, assessed whether *Salmonella* adopted a similar residence lifestyle in our CAM tumor model. Based on the percentage of the total tumor-associated bacterial population, approximately 99% of bacteria were extracellular, with ~1% of the bacteria found inside the CAM tumor cells (WT, *ΔhilD*, and *ΔssrB*) (Fig. 5C). Hence, this finding corroborated that the loss of SPI-1 and SPI-2 genes did not impair the tumor-colonizing ability of STm (Fig. 5B), because STm was predominantly extracellular on the CAM tumors.

To address the question of whether extracellular STm also forms biofilms on CAM tumors, we infected tumors with *Salmonella* WT or a *ΔcsgD* null strain; CsgD is the master biofilm regulator (35). The extracellular bacterial load recovered indicated that both strains successfully colonized the tumor to the same extent: a mean log CFU/g of ~7 to 8, at 1 and 3 DPI (Fig. 5D). Thus, in contrast to the mouse tumor model (34), the ability to form biofilms was not required for the extracellular accumulation of STm on CAM tumors.

### How does the clinical strain VNP2009 compare with WT *Salmonella*?

Despite promising results in xenograft mouse models (effective tumor colonization with tumor growth retardation), the clinical strain VNP20009 did not perform well (low bacterial tumor colonization) in a human clinical trial. It was therefore of interest to evaluate colonization by VNP20009 compared to its parental WT strain (15, 36) in both 3D tumor spheroid and CAM tumor models.

### The clinical strain VNP20009 replicates poorly within 3D tumor spheroids

**Fig 6:**
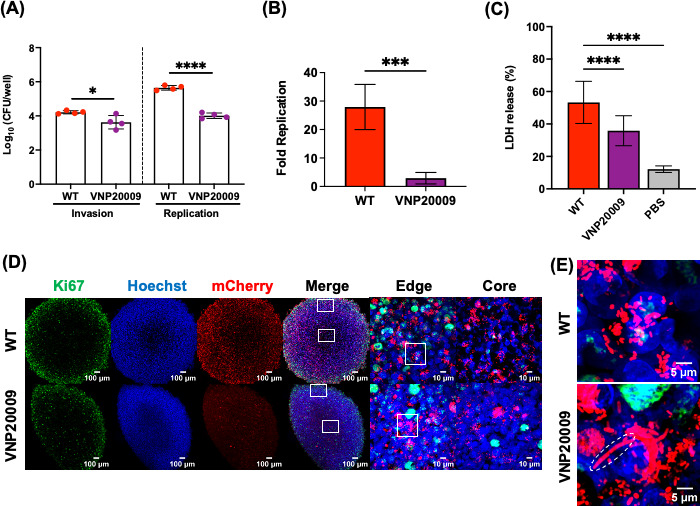
Replication of VNP20009 within 3D tumor spheroids is impaired. (**A**) Intracellular bacterial enumeration from WT-infected and VNP20009-infected tumor spheroids, measured at invasion (mean log CFU/well at 2 HPI: WT = 4.2, VNP20009 = 3.6) and replication (mean log CFU/well at 16 HPI: WT = 5.6, VNP20009 = 4) stages of infection. Dots: biological replicates, each with a minimum of five technical replicates. The bars represent the mean ± SD. The statistical significance was determined by unpaired *t*-test. (**B**) Fold replication (16 HPI/2 HPI) of WT (28-fold) and VNP20009 (threefold) strains within tumor spheroids. The bars represent the means of four biological replicates, a minimum of five technical replicates each. Error bars = ±SD. The statistical significance was determined by unpaired *t*-test. (**C**) LDH release from tumor spheroids infected with WT (53%) and VNP20009 (36%) at 16 HPI. The bars represent the means of three biological replicates, a minimum of five technical replicates each. Error bars = ±SD. Statistical significance was determined by one-way ANOVA with Sidak’s multiple comparison test. For all, non-significant (ns), *P ≤ 0.05, **P ≤ 0.01, ***P ≤ 0.001, and ****P ≤ 0.0001. (**D**) Representative immunofluorescence images of 3D tumor spheroids with Ki47 AlexaFluor 488 (green), Hoechst (blue), and mCherry-tagged bacterial strains (red) at 10× and 100× objectives. Maximum intensity projections of Z-stack images were taken with an Olympus Super Resolution Spinning Disk SpinSR-10 microscope. The representative image of a 3D tumor spheroid infected with WT *Salmonella* (Fig. 4D) was used to compare with a representative image of a tumor spheroid infected with the clinical strain VNP20009. (**E**) Zoomed 300% magnification of white box ROI from 100× tumor spheroids edge of both WT and VNP20009 to compare and visualize the cell morphology defect of VNP20009, outlined by dotted lines.

We first investigated the performance of the clinical strain VNP20009 within 3D tumor spheroids. Compared to WT, the VNP20009 strain displayed a modest invasion defect with a mean reduction of 0.6 log CFU/well (Fig. 6A, left columns). However, the replication defect at 16 HPI was striking, with an overall mean reduction of 1.6 log CFU/well relative to WT (Fig. 6A, right columns). Replication of the WT was 28-fold, but VNP20009 only increased threefold (Fig. 6B). These differences indicated a stark difference in the ability of the attenuated VNP20009 strain to grow within the tumor spheroid compared to the WT. The possibility that differential growth rates between strains contributed to tumor invasion was ruled out, as the growth rate of VNP20009 was not compromised under bacterial culture conditions (Fig. S3). Furthermore, bacterial inoculums were prepared under the same SPI-1-inducing condition for both strains. The percentage of bacteria that were SPI-1 positive (using a P*prgH*-mCherry reporter) for WT and VNP20009 was similar at 67% and 62%, respectively, of the total population (Fig. S4). We next compared the ability of VNP20009 to induce tumor cell cytotoxicity with the LDH assay after 16 HPI. WT-infected tumor spheroids released higher amounts of LDH (53%) than VNP20009 (36%) (Fig. 6C).

Representative images of VNP20009-infected tumor spheroids highlighted the reduction in intracellular bacteria compared to WT (Fig. 6D), corroborating the bacterial enumeration data set (Fig. 6A and B). Furthermore, tumor spheroids infected with VNP20009 revealed distinct cell morphological defects of the strain with elongated bacterial cells (Fig. 6E), confirming the strain growth defects within the tumor spheroid (see Discussion).

### Reduced CAM tumor colonization of VNP20009 compromises its anti-tumor immunity

**Fig 7:**
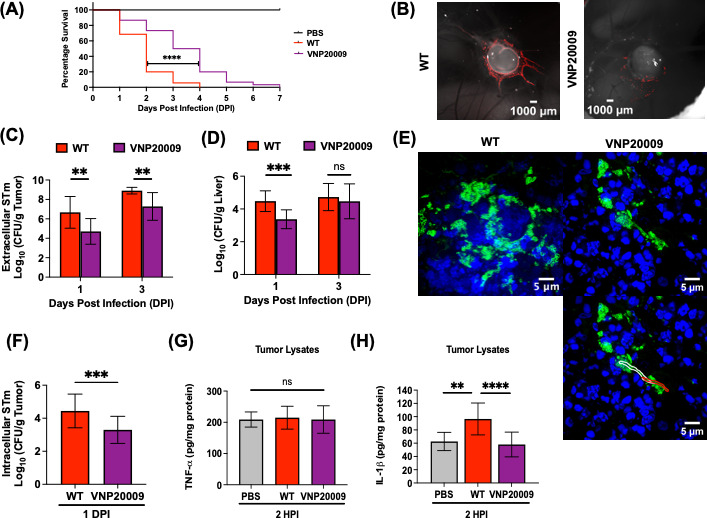
Reduced tumor colonization by VNP20009 impacts its anti-tumor immunity. (**A**) Tumor-bearing chick embryos infected with VNP20009 (purple line) showed significant improvement in the percentage survival as well as longer survival times compared to chick embryos that were infected with WT (red line) at a dose of ~50 CFU. For survival analysis, the log-rank (Mantel-Cox) test was used. (**B**) Representative live, *in ovo* images of mCherry-tagged *Salmonella*, WT, and VNP20009 colonizing CAM tumors at 1 DPI. (**C and D**) Extracellular *Salmonella* WT and VNP20009 CFU recovered per gram of tumor and liver at 1 and 3 DPI. The statistical significance was determined by unpaired *t*-test. (**E**) Super Resolution 320× magnified representative images of WT compared with distinct cell morphology defects of VNP20009 observed within CAM tumor tissue sections at 3 DPI, LPS (green), and Hoechst (blue). Elongated bacteria cells are outlined with white and red lines. (**F**) Intracellular *Salmonella* WT and VNP20009 CFU recovered per gram of tumor at 1 DPI. The statistical significance was determined by unpaired *t*-test. (**G and H**) Tumor necrosis factor-alpha (TNF-α) and interleukin-1 beta (IL-1β) expression level in tumor lysates at 2 HPI, measured by enzyme-linked immunosorbent assay, and data were expressed as pg per mg of total protein. The results represent the mean ± SD. Statistical significance was determined by one-way ANOVA with Sidak’s multiple comparison test. Not significant (ns), *P ≤ 0.05, **P ≤ 0.01, ***P ≤ 0.001, and ****P ≤ 0.0001. *N* = 2 with 5–10 tumor-bearing chick embryos per group.

We examined the tumor colonization ability of VNP2009 in the CAM tumor model. The effect of VNP20009 administration (~50 CFU) on chick embryo mortality was first evaluated against the WT and PBS control groups. A significant improvement in survival rate and prolonged survival time was noted for tumor-bearing chick embryos infected with VNP20009 compared to the WT (Fig. 7A). Chick embryos completely succumbed to WT infection by 4 days, whereas VNP-infected embryos survived for up to 7 days. We next assessed the tumor-colonizing capability of VNP20009. Live *in ovo* images of mCherry-tagged bacterial strains colonizing the CAM tumor highlighted the marked reduction in fluorescence signals of VNP20009 at 1 DPI (Fig. 7B) compared to the WT. The image emphasizes the strain attenuation of VNP2009 in tumor colonization, which was also apparent when we examined the intra-tumoral distribution (Fig. S5). Compared to WT, VNP20009 was remarkably reduced throughout the tumor tissue sections at all depths. This result was further confirmed by an overall reduction in the extracellular bacterial load recovered from VNP20009-infected tumors (Fig. 7C). At 1 DPI, the extracellular bacterial load of VNP20009 was only ~1% of WT. A reduction of VNP20009 tumor colonization was still evident at 3 DPI and was ~36% of WT colonization (Fig. 7C). When we compared the two strains and their ability to colonize the liver (Fig. 7D), VNP2009 exhibited an early defect in dissemination (~10% of WT). However, the attenuated strain was able to colonize chick embryo livers to a similar level as the WT by 3 DPI (Fig. 7D). This was in contrast to tumor colonization (Fig. 7C), where the rate of VNP2009 colonization remained weaker than the WT over time. Furthermore, a cell morphological defect was observed, as elongated chains of VNP20009 were detected in CAM tumor tissue sections at 3 DPI (Fig. 7E). These chains were strikingly similar to our previous observations in 3D tumor spheroids (Fig. 6E). The CAM tumor invasion rate of VNP20009 was also compromised compared to the WT invasion rate (see intracellular bacterial counts, Fig. 7F).

Chick embryos possess only partial immunity at early developmental stages (before ED9), but as immunocompetence develops, embryos are capable of mounting an inflammatory response with cytokine expression from host immune cells (37). Induction of pro-inflammatory cytokines, such as tumor necrosis factor-alpha (TNF-α) and interleukin-1 beta (IL-1β), following *Salmonella* infection in tumor-bearing hosts is indicative of host-mediated tumoricidal effects (38). To investigate whether host immune factors play an indirect role in driving anti-tumor immunity, CAM tumors were infected with WT, VNP20009, or PBS at ED11, and cytokine expression in tumor lysates at 2 HPI was measured. TNF-α levels were similar across uninfected and infected tumors (WT and VNP20009) at 2 HPI (Fig. 7G). In contrast, IL-1β was highly expressed in WT-infected tumors, while IL-1β levels were similar between uninfected (PBS) and VNP20009-infected tumors at 2 HPI (Fig. 7H). This result suggests that reduced tumor colonization by VNP20009 failed to trigger the activation of host-mediated anti-tumor immunity in the CAM model.

## DISCUSSION

To the best of our knowledge, this is the first study to exploit the CAM tumor model as an *in vivo* model to study *Salmonella*-mediated anticancer therapy. Our work established that the chick embryo CAM model reproduces key features of tumor-targeting STm reported in the mouse model: (i) tumor specificity in colonization (Fig. 2A), (ii) intra-tumoral distribution (Fig. 2B), (iii) direct induction of tumor cell death ( Fig. 2C through G), and (iv) indirect stimulation of anti-tumor immunity to some degree in the partial immunocompetent host (Fig. 7H). To assess whether the CAM model recapitulated the published mouse studies, *Salmonella* key pathogenic traits were investigated for their respective roles in tumor colonization and invasion.

Flagellar-dependent motility as a driver of bacterial migration, tumor colonization, and intra-tumoral distribution was not essential, as a non-motile *Salmonella* (Δ*flgK*) was not impaired for colonization in the CAM model (Fig. 3). This finding agrees with published mouse data indicating that tumor colonization by *Salmonella* is a passive process (not requiring bacterial motility), where bacterial infection induces a cytokine storm and hemorrhage, facilitating the deposition of bacteria at the tumor site (23, 39). In the CAM tumor model, the passive tumor migration process of STm (via a distant inoculation route, Fig. 3A and C) was most likely aided by the active movement of a live chick embryo just beneath the CAM layer.

In our *in vitro* 3D tumor spheroids, both SPI-1 and SPI-2 contributed to STm intracellular replication within the tumor spheroids (Fig. 4). In the mouse model, there are conflicting reports as to whether SPI-1 or SPI-2 virulence genes are required for tumor invasion. Although SPI-1-mediated tumor invasion (3) and SPI-2-associated intra-tumoral replication (40) were reported to be important, in the majority of mouse tumor studies, virulence functions were not required, as bacteria were extracellular on tumor sites (23, 34, 41, 42). Consistent with these latter findings, virulence functions were not required for CAM tumor colonization and invasion (Fig. 5A and B). In our experiments, *Salmonella* strains (WT, Δ*hilD*, and Δ*ssrB*) were predominately extracellular on the CAM tumor (Fig. 5C). The inconsistency between *in vitro* and *in ovo* data is likely attributed to the fact that 3D spheroids consist of a single cancer cell type, which lacks the heterogeneity and vascularization of the chick CAM model to mimic the complexity of the tumor microenvironment within the host and its interaction with bacteria.

In the mouse model, an almost exclusive extracellular residence as biofilms on solid tumors was reported to be a bacterial protective mechanism against phagocytosis by host immune cells, specifically neutrophils (34). However, the CAM tumor model did not reproduce the STm biofilm phenotype, as the Δ*csgD* strain was able to colonize tumors to similar levels as WT (Fig. 5D). Immunological differences in the tumor microenvironment between the two models (mouse vs chick embryos) might explain the differences in an adaptation of bacterial lifestyles on solid tumors. In contrast to a mammalian host, chick embryos have heterophils, the avian equivalent of neutrophils, but lack myeloperoxidase (37). Previously it was shown that heterophil recruitment to the inflammation site following lipopolysaccharide stimulation was detectible at ED7 (43), but fully functional heterophils within developing embryos were only described at ED18, 3 days before hatching (44, 45). Our CAM experimental timeline is completed by ED14 due to the low survival percentage of tumor-bearing chick embryos at an STm infectious dose of ~50 CFU. Therefore, the lack of functional heterophils within the chick embryonic immune system in the current study timeline likely influences the non-biofilm residence of STm on CAM tumors. As with every model, the CAM tumor model has its limitations. As highlighted above, the lack of a functional host immune system and the short experimental timeline (inability to assess STm impact on tumor growth or regression) should be considered during experimental design and data interpretation.

Previous studies examining the anti-tumor efficacy of the highly attenuated STm strain VNP20009 failed to directly compare the tumor-colonizing level of the strain relative to its parental WT (15, 16, 18, 46–48). Here, we used both the *in vitro* 3D tumor spheroids and CAM tumor model to provide evidence of reduced tumor colonization, invasion, and replication by VNP20009 compared to WT (Fig. 6A, B, 7B, C and F). In 3D tumor spheroids, the reduced intracellular replication of VNP20009 has consequences that limit its ability to kill tumor cells (Fig. 6C). In addition, we observed cell morphology defects of VNP20009 (i.e., elongated bacterial cells) in both models during infection (Fig. 6E and 7E). Although deletion of *msbB* in VNP20009 has been reported to exhibit severe growth and morphology defects under *in vitro* growth conditions in the presence of 5% CO_2_, acidic pH, and high osmolarity (49, 50), our work provides additional evidence of the inherent growth sensitivity of VNP20009 in the *in vivo* tumor microenvironment (Fig. 7E). We reason that this growth defect is related to cancer metabolism, which undergoes aerobic glycolysis (also known as the Warburg effect), resulting in elevated CO_2_ levels and lactic acid in the tumor microenvironment (51).

A poor human clinical outcome of VNP20009 was mainly the result of low tumor colonization that compromised its anti-tumor efficacy (20). In our CAM tumor model, we showed that IL-1β expression levels in tumor lysates of VNP20009 and PBS controls were similar (Fig. 7H), suggesting that the reduced presence of VNP20009 on tumors was insufficient to elicit the anti-tumor immune response in the CAM model. These results highlighted the importance of maximizing the tumor-colonizing capability of the bacterial strain as a primary factor in strengthening its anti-tumor effectiveness.

In summary, the chick embryo CAM model offers a valuable tool for initial screening and preclinical assessment of tumor-colonizing *Salmonella* strains. Its accessibility, vascularization, and relevance to tumor biology make it a suitable choice for studying bacterial interactions with tumors and assessing potential therapeutic strains for bacterial-mediated cancer therapy.

## MATERIALS AND METHODS

### 3D spheroid infection model

A549 tumor cell spheroids were formed with the 96-well bioprinting kit (Greiner Bio-One, catalog no. 655841) according to the manufacturer’s guidelines. Cells were first magnetized with NanoShuttle-PL, nanoparticles overnight. Magnetized cells were counted and seeded at the same cell density of ~50,000 cells/well. Using the 96-well magnetic drive placed at the bottom of the well, magnetized cells were printed into spheroid for 24 hours. The next day, STm strains grown under SPI-1-inducing conditions were added into each well at a multiplicity of infection of 100. Bacteria were added for 30 minutes, high gentamicin concentrations of 100 µg/mL were added for 1.5 hours for invasion (2 HPI) and changed to low gentamicin of 20 µg/mL for the remainder of the infection process. We confirmed that all extracellular bacteria were eliminated post-gentamicin treatment by plating the cell culture medium. At each step of the cell media replacement and wash process, the 96-well plate was placed on the holding drive to hold the spheroids down while aspirating the solution to prevent cell loss. At both 2 HPI (invasion) and 16 HPI (replication), cell media were aspirated, washed with phosphate-buffered saline, PBS (Roche, 11666789001), and replaced with 1% Triton X-100 (Sigma, T8787-100ml) for 10 minutes to lyse the cells. Spheroids were homogenized by the vigorous pipetting method. Lysates were then serially diluted and plated for intracellular bacterial enumeration.

### Whole-mount immunofluorescence staining of 3D tumor spheroids

At the end of a 16 hours STm infection, tumor spheroids were fixed in 4% paraformaldehyde for ~5 hours, washed with PBS thrice, and then processed for whole-mount immunofluorescence staining. Each tumor spheroid was individually picked up using the magpen with teflon caps (catalog no. 657850, Grenier Bio-One), during each step of the staining process for solution replacement. Antigen retrieval was performed by transferring spheroids into a citrate buffer (Sigma, C9999-100ml) solution for 20 minutes in 99°C water bath before allowing the solution to cool to room temperature. The permeabilization step was performed with 0.3% Triton-X/PBS for 15 minutes followed by incubation with blocking buffer and 5% bovine serum albumin in PBS (Sigma-Aldrich, catalog no. A7030) for 1 hour. Spheroids were then incubated with primary antibody, recombinant anti-Ki67 (Abcam, catalog no. ab16667) diluted in blocking solution (1:200), overnight at 4°C in the dark. The next day, spheroids were incubated with a secondary antibody to Ki67, Alexa Fluor 488 goat anti-rabbit IgG H&L (Abcam, catalog no. ab150081), and Hoechst (blue, Invitrogen, H3570) for 1 hour. Whole spheroids were then mounted onto the slide with a prolong gold antifade mountant (Invitrogen, catalog no. P36934), coverslip sealed with nail polish, and dried overnight in the dark. Whole-mount spheroids were imaged with Olympus SpinSR-10 spinning disk confocal at 10× air objective (NA 0.40) and 100× oil objective (NA 1.5). Acquired microscopy images were processed with Fiji software (25).

### Embryonated chicken CAM tumor model

Specific pathogen-free (SPF), premium fertilized chicken eggs were purchased from AVS bio and set horizontally in a Rcom Max 50 incubator at 37°C and 60% humidity. On ED3, CAM of the chick embryo was dropped by inserting a thin needle at the apex of the egg and removing ~4 mL of albumin with a syringe and 21G needle. A small window (~1 cm^2^) was made on top of the eggshell and resealed with 3M Tegaderm transparent film dressing. On ED7, A549 cell pellets (~2 × 10^6^ cells/egg) were mixed with 50 µL Matrigel (Corning 354234 or 354263) and were grafted into the center of a sterilized silicone ring placed on top of the CAM layer. Eggshell windows were then re-sealed with Tegaderm and put back into the incubator to allow tumor growth for four days. On ED11, the silicone ring was first carefully removed from the CAM without disrupting the established tumor before the application of the bacterial inoculum directly onto the tumor with a pipette. Bacterial inoculums were plated each time to confirm that a similar dose was applied for all strains. For the distant inoculation route experiment, bacteria were deposited onto a sterile cut-out filter paper, placed a short distance away from the tumor (~0.5 cm). At different time points, tumors and livers were harvested for the enumeration of viable bacteria by homogenization, serial dilution, and plating methods. All chick embryo experiments were repeated twice (*N* = 2) with 5–10 tumor-bearing embryos in each treated group.

### TUNEL assay and apoptosis quantification with Fiji

Detection of apoptotic cells on tumor tissue sections was performed with Click-iT Plus TUNEL assay (Invitrogen, Thermo Fisher Scientific Lot 2559160) according to the manufacturer’s guidelines. Immunofluorescence images were acquired with an Olympus Super Res Spinning Disk, SpinSR-10 microscope at 100× oil objective (NA 1.5). Quantification of TUNEL-positive nuclei as well as Hoechst nuclei was performed with Fiji (25) following the optimized and unbiased image processing protocol. Briefly, images were first split into separate color channels and compressed with *z*-axis maximum intensity projection. Background noise reduction was performed by subtracting background values from each channel. Images were then converted to grayscale with 8-bit. The binarization thresholding function available in Fiji (25) was utilized, and thresholding algorithms that capture the best outline of stained cells for each channel were selected (52). Moments and Li auto-thresholding algorithms were opted for highlighting TUNEL-positive nuclei and Hoechst-positive cells, respectively. Lastly, the analyze particle function was applied for the count read-outs. The percentage of TUNEL-stained cells was calculated against the total cell numbers (Hoechst-positive cells) within the same region.

### Statistical analysis

All statistical analyses were performed with GraphPad Prism 10.2.1.

## SUPPLEMENTAL MATERIAL

Supplemental Material mbio.03590-24-s0001.docx Supplemental table, figure legends, and additional experimental details.

Fig S1 mbio.03590-24-s0002.tiff Optimal infectious dose of STm for the CAM tumor model.

Fig S2 mbio.03590-24-s0003.tiff CAM tumor sections infected with WT at 3DPI.

Fig S3 mbio.03590-24-s0004.tiff Growth curve of all bacterial strains.

Fig S4 mbio.03590-24-s0005.tiff SPI-1 expression in STm WT and VNP20009 strains.

Fig S5 mbio.03590-24-s0006.tiff CAM tumor sections infected with VNP20009 at 1- and 3-DPI.

ASM does not own the copyrights to Supplemental Material that may be linked to, or accessed through, an article. The authors have granted ASM a non-exclusive, world-wide license to publish the Supplemental Material files. Please contact the corresponding author directly for reuse.

## References

[1] Pawelek JM, Low KB, Bermudes D. 1997. Tumor-targeted Salmonella as a novel anticancer vector. Cancer Res 57:4537–4544. 9377566

[2] Leschner S, Weiss S. 2010. Salmonella-allies in the fight against cancer. J Mol Med (Berl) 88:763–773. doi:10.1007/s00109-010-0636-z 20526574

[3] Avogadri F, Martinoli C, Petrovska L, Chiodoni C, Transidico P, Bronte V, Longhi R, Colombo MP, Dougan G, Rescigno M. 2005. Cancer immunotherapy based on killing of Salmonella-infected tumor cells. Cancer Res 65:3920–3927. doi:10.1158/0008-5472.CAN-04-3002 15867392

[4] Kaimala S, Al-Sbiei A, Cabral-Marques O, Fernandez-Cabezudo MJ, Al-Ramadi BK. 2018. Attenuated bacteria as immunotherapeutic tools for cancer treatment. Front Oncol 8:136. doi:10.3389/fonc.2018.00136 29765907 PMC5938341

[5] Saccheri F, Pozzi C, Avogadri F, Barozzi S, Faretta M, Fusi P, Rescigno M. 2010. Bacteria-induced gap junctions in tumors favor antigen cross-presentation and antitumor immunity. Sci Transl Med 2:44ra57. doi:10.1126/scitranslmed.3000739 20702856

[6] Zhou S, Gravekamp C, Bermudes D, Liu K. 2018. Tumour-targeting bacteria engineered to fight cancer. Nat Rev Cancer 18:727–743. doi:10.1038/s41568-018-0070-z 30405213 PMC6902869

[7] Giannella RA. 1996. *Salmonella*. In Baron S (ed), Medical microbiology, 4th ed. University of Texas Medical Branch at Galveston, Galveston (TX). 21413252

[8] Sys G, Van Bockstal M, Forsyth R, Balke M, Poffyn B, Uyttendaele D, Bracke M, De Wever O. 2012. Tumor grafts derived from sarcoma patients retain tumor morphology, viability, and invasion potential and indicate disease outcomes in the chick chorioallantoic membrane model. Cancer Lett 326:69–78. doi:10.1016/j.canlet.2012.07.023 22841668

[9] Kain KH, Miller JWI, Jones‐Paris CR, Thomason RT, Lewis JD, Bader DM, Barnett JV, Zijlstra A. 2014. The chick embryo as an expanding experimental model for cancer and cardiovascular research. Dev Dyn 243:216–228. doi:10.1002/dvdy.24093 24357262 PMC4164046

[10] Mitrevska K, Merlos Rodrigo MA, Cernei N, Michalkova H, Splichal Z, Hynek D, Zitka O, Heger Z, Kopel P, Adam V, Milosavljevic V. 2023. Chick chorioallantoic membrane (CAM) assay for the evaluation of the antitumor and antimetastatic activity of platinum-based drugs in association with the impact on the amino acid metabolism. Mater Today Bio 19:100570. doi:10.1016/j.mtbio.2023.100570 PMC9941372 36824411

[11] Miebach L, Berner J, Bekeschus S. 2022. In ovo model in cancer research and tumor immunology. Front Immunol 13. doi:10.3389/fimmu.2022.1006064 PMC9556724 36248802

[12] Ishihara M, Hu J, Wong A, Cano-Ruiz C, Wu L. 2019. Mouse- and patient-derived CAM xenografts for studying metastatic renal cell carcinoma. Enzymes 46:59–80. doi:10.1016/bs.enz.2019.08.009 31727277

[13] Fischer D, Fluegen G, Garcia P, Ghaffari-Tabrizi-Wizsy N, Gribaldo L, Huang RY-J, Rasche V, Ribatti D, Rousset X, Pinto MT, Viallet J, Wang Y, Schneider-Stock R. 2022. The CAM model-Q&A with experts. Cancers (Basel) 15:191. doi:10.3390/cancers15010191 36612187 PMC9818221

[14] Mon KKZ, Si Z, Chan-Park MB, Kenney LJ. 2022. Polyimidazolium protects against an invasive clinical isolate of Salmonella typhimurium. Antimicrob Agents Chemother 66:e0059722. doi:10.1128/aac.00597-22 36094258 PMC9578408

[15] Low KB, Ittensohn M, Luo X, Zheng L-M, King I, Pawelek JM, Bermudes D. 2004. Edited by C. J. Springer. Suicide gene therapy methods and reviews, p 47–59. Humana Press, Totowa, NJ.

[16] Clairmont C, Lee KC, Pike J, Ittensohn M, Low KB, Pawelek J, Bermudes D, Brecher SM, Margitich D, Turnier J, Li Z, Luo X, King I, Zheng LM. 2000. Biodistribution and genetic stability of the novel antitumor agent VNP20009, a genetically modified strain of Salmonella typhimurium. J Infect Dis 181:1996–2002. doi:10.1086/315497 10837181

[17] Low KB, Ittensohn M, Le T, Platt J, Sodi S, Amoss M, Ash O, Carmichael E, Chakraborty A, Fischer J, Lin SL, Luo X, Miller SI, Zheng L, King I, Pawelek JM, Bermudes D. 1999. Lipid A mutant Salmonella with suppressed virulence and TNFalpha induction retain tumor-targeting in vivo. Nat Biotechnol 17:37–41. doi:10.1038/5205 9920266

[18] Luo X, Li Z, Lin S, Le T, Ittensohn M, Bermudes D, Runyab JD, Shen S, Chen J, King IC, Zheng L. 2001. Antitumor effect of VNP20009, an attenuated Salmonella, in murine tumor models. Oncol Res Featur Preclin Clin Cancer Ther 12:501–508. doi:10.3727/096504001108747512 11939414

[19] King I, Luo X, Feng M, Ittensohn M, Li Z, Belcourt M, Lin S, Le T, Pike J, Troy K, Sznol M, Clairmont C, Bermudes D, Zheng L-M. 2000. Tumour therapy using Salmonella. Emerg Drugs 5:211–219. doi:10.1517/14728214.5.2.211

[20] Toso JF, Gill VJ, Hwu P, Marincola FM, Restifo NP, Schwartzentruber DJ, Sherry RM, Topalian SL, Yang JC, Stock F, Freezer LJ, Morton KE, Seipp C, Haworth L, Mavroukakis S, White D, MacDonald S, Mao J, Sznol M, Rosenberg SA. 2002. Phase I study of the intravenous administration of attenuated Salmonella typhimurium to patients with metastatic melanoma. J Clin Oncol 20:142–152. doi:10.1200/JCO.2002.20.1.142 11773163 PMC2064865

[21] Tseng H, Gage JA, Raphael RM, Moore RH, Killian TC, Grande-Allen KJ, Souza GR. 2013. Assembly of a three-dimensional multitype bronchiole coculture model using magnetic levitation. Tissue Eng Part C Methods 19:665–675. doi:10.1089/ten.TEC.2012.0157 23301612

[22] Zanoni M, Piccinini F, Arienti C, Zamagni A, Santi S, Polico R, Bevilacqua A, Tesei A. 2016. 3D tumor spheroid models for in vitro therapeutic screening: a systematic approach to enhance the biological relevance of data obtained. Sci Rep 6:19103. doi:10.1038/srep19103 26752500 PMC4707510

[23] Crull K, Bumann D, Weiss S. 2011. Influence of infection route and virulence factors on colonization of solid tumors by Salmonella enterica serovar Typhimurium. FEMS Immunol Med Microbiol 62:75–83. doi:10.1111/j.1574-695X.2011.00790.x 21314734

[24] Wang C-Z, Kazmierczak RA, Eisenstark A. 2016. Strains, mechanism, and perspective: Salmonella-based cancer therapy. Int J Microbiol 2016:5678702. doi:10.1155/2016/5678702 27190519 PMC4848419

[25] Schindelin J, Arganda-Carreras I, Frise E, Kaynig V, Longair M, Pietzsch T, Preibisch S, Rueden C, Saalfeld S, Schmid B, Tinevez J-Y, White DJ, Hartenstein V, Eliceiri K, Tomancak P, Cardona A. 2012. Fiji: an open-source platform for biological-image analysis. Nat Methods 9:676–682. doi:10.1038/nmeth.2019 22743772 PMC3855844

[26] Toley BJ, Forbes NS. 2012. Motility is critical for effective distribution and accumulation of bacteria in tumor tissue. Integr Biol (Camb) 4:165–176. doi:10.1039/c2ib00091a 22193245 PMC4956405

[27] Thornlow DN, Brackett EL, Gigas JM, Van Dessel N, Forbes NS. 2015. Persistent enhancement of bacterial motility increases tumor penetration. Biotechnol Bioeng 112:2397–2405. doi:10.1002/bit.25645 25976712 PMC4586311

[28] Coutermarsh-Ott SL, Broadway KM, Scharf BE, Allen IC. 2017. Effect of Salmonella enterica serovar Typhimurium VNP20009 and VNP20009 with restored chemotaxis on 4T1 mouse mammary carcinoma progression. Oncotarget 8:33601–33613. doi:10.18632/oncotarget.16830 28431394 PMC5464893

[29] Homma M, DeRosier DJ, Macnab RM. 1990. Flagellar hook and hook-associated proteins of Salmonella typhimurium and their relationship to other axial components of the flagellum. J Mol Biol 213:819–832. doi:10.1016/S0022-2836(05)80266-9 2193164

[30] Cirillo DM, Valdivia RH, Monack DM, Falkow S. 1998. Macrophage-dependent induction of the Salmonella pathogenicity island 2 type III secretion system and its role in intracellular survival. Mol Microbiol 30:175–188. doi:10.1046/j.1365-2958.1998.01048.x 9786194

[31] Main-Hester KL, Colpitts KM, Thomas GA, Fang FC, Libby SJ. 2008. Coordinate regulation of Salmonella pathogenicity island 1 (SPI1) and SPI4 in Salmonella enterica serovar Typhimurium. Infect Immun 76:1024–1035. doi:10.1128/IAI.01224-07 18160484 PMC2258849

[32] Haraga A, Ohlson MB, Miller SI. 2008. Salmonellae interplay with host cells. Nat Rev Microbiol 6:53–66. doi:10.1038/nrmicro1788 18026123

[33] Shetty D, Kenney LJ. 2023. A pH-sensitive switch activates virulence in Salmonella. Elife 12:e85690. doi:10.7554/eLife.85690 37706506 PMC10519707

[34] Crull K, Rohde M, Westphal K, Loessner H, Wolf K, Felipe-López A, Hensel M, Weiss S. 2011. Biofilm formation by Salmonella enterica serovar Typhimurium colonizing solid tumours. Cell Microbiol 13:1223–1233. doi:10.1111/j.1462-5822.2011.01612.x 21507181

[35] Desai SK, Winardhi RS, Periasamy S, Dykas MM, Jie Y, Kenney LJ. 2016. The horizontally-acquired response regulator SsrB drives a Salmonella lifestyle switch by relieving biofilm silencing. Elife 5:e10747. doi:10.7554/eLife.10747 26880544 PMC4769171

[36] Low KB, Ittensohn M, Luo X, Zheng L-M, King I, Pawelek JM, Bermudes D. 2004. Construction of VNP20009: a novel, genetically stable antibiotic-sensitive strain of tumor-targeting Salmonella for parenteral administration in humans. Methods Mol Med 90:47–60. 14657558

[37] Garcia P, Wang Y, Viallet J, Macek Jilkova Z. 2021. The chicken embryo model: a novel and relevant model for immune-based studies. Front Immunol 12:791081. doi:10.3389/fimmu.2021.791081 34868080 PMC8640176

[38] Kim J-E, Phan TX, Nguyen VH, Dinh-Vu H-V, Zheng JH, Yun M, Park S-G, Hong Y, Choy HE, Szardenings M, Hwang W, Park J-A, Park S, Im S-H, Min J-J. 2015. Salmonella typhimurium suppresses tumor growth via the pro-inflammatory cytokine interleukin-1β. Theranostics 5:1328–1342. doi:10.7150/thno.11432 26516371 PMC4615736

[39] Leschner S, Westphal K, Dietrich N, Viegas N, Jablonska J, Lyszkiewicz M, Lienenklaus S, Falk W, Gekara N, Loessner H, Weiss S. 2009. Tumor invasion of Salmonella enterica serovar Typhimurium is accompanied by strong hemorrhage promoted by TNF-alpha. PLoS ONE 4:e6692. doi:10.1371/journal.pone.0006692 19693266 PMC2724709

[40] Pawelek JM, Sodi S, Chakraborty AK, Platt JT, Miller S, Holden DW, Hensel M, Low KB. 2002. Salmonella pathogenicity island-2 and anticancer activity in mice. Cancer Gene Ther 9:813–818. doi:10.1038/sj.cgt.7700501 12224021

[41] Loessner H, Endmann A, Leschner S, Westphal K, Rohde M, Miloud T, Hämmerling G, Neuhaus K, Weiss S. 2007. Remote control of tumour-targeted Salmonella enterica serovar typhimurium by the use of L-arabinose as inducer of bacterial gene expression in vivo. Cell Microbiol 9:1529–1537. doi:10.1111/j.1462-5822.2007.00890.x 17298393

[42] Silva-Valenzuela CA, Desai PT, Molina-Quiroz RC, Pezoa D, Zhang Y, Porwollik S, Zhao M, Hoffman RM, Contreras I, Santiviago CA, McClelland M. 2016. Solid tumors provide niche-specific conditions that lead to preferential growth of Salmonella. Oncotarget 7:35169–35180. doi:10.18632/oncotarget.9071 27145267 PMC5085218

[43] Valdes TI, Kreutzer D, Moussy F. 2002. The chick chorioallantoic membrane as a novel in vivo model for the testing of biomaterials. J Biomed Mater Res 62:273–282. doi:10.1002/jbm.10152 12209948

[44] Kogut MH, Lowry VK, Moyes RB, Bowden LL, Bowden R, Genovese K, Deloach JR. 1998. Lymphokine-augmented activation of avian heterophils. Poult Sci 77:964–971. doi:10.1093/ps/77.7.964 9657605

[45] Kogut MH, Holtzapple C, Lowry VK, Genovese K, Stanker LH. 1998. Functional responses of neonatal chicken and turkey heterophils following stimulation by inflammatory agonists. Am J Vet Res 59:1404–1408. 9829397

[46] Li M, Lu M, Lai Y, Zhang X, Li Y, Mao P, Liang Z, Mu Y, Lin Y, Zhao AZ, Zhao Z, Zhou S, Li F. 2020. Inhibition of acute leukemia with attenuated Salmonella typhimurium strain VNP20009. Biomed Pharmacother 129:110425. doi:10.1016/j.biopha.2020.110425 32570123

[47] Liu X, Guo Y, Sun Y, Chen Y, Tan W, Min J-J, Zheng JH. 2022. Comparison of anticancer activities and biosafety between Salmonella enterica serovar Typhimurium ΔppGpp and VNP20009 in a murine cancer model. Front Microbiol 13:914575. doi:10.3389/fmicb.2022.914575 35847095 PMC9277105

[48] Ahmed SG, Oliva G, Shao M, Wang X, Mekalanos JJ, Brenner GJ. 2022. Intratumoral injection of schwannoma with attenuated Salmonella typhimurium induces antitumor immunity and controls tumor growth. Proc Natl Acad Sci U S A 119:e2202719119. doi:10.1073/pnas.2202719119 35675425 PMC9214496

[49] Karsten V, Murray SR, Pike J, Troy K, Ittensohn M, Kondradzhyan M, Low KB, Bermudes D. 2009. msbB deletion confers acute sensitivity to CO2 in Salmonella enterica serovar typhimurium that can be suppressed by a loss-of-function mutation in zwf. BMC Microbiol 9:170. doi:10.1186/1471-2180-9-170 19689794 PMC2745414

[50] Murray SR, Bermudes D, de Felipe KS, Low KB. 2001. Extragenic suppressors of growth defects in msbB Salmonella. J Bacteriol 183:5554–5561. doi:10.1128/JB.183.19.5554-5561.2001 11544217 PMC95446

[51] Vander Heiden MG, Cantley LC, Thompson CB. 2009. Understanding the warburg effect: the metabolic requirements of cell proliferation. Science 324:1029–1033. doi:10.1126/science.1160809 19460998 PMC2849637

[52] de Noiron J, Hoareau M, Colin J, Guénal I. 2021. Apoptosis quantification in tissue: development of a semi-automatic protocol and assessment of critical steps of image processing. Biomolecules 11:1523. doi:10.3390/biom11101523 34680157 PMC8533694

