# Supplementary figures and images for: Evaluation of tumor-colonizing Salmonella strains using the chick chorioallantoic membrane model
Source: mBio. 2025 Jan 28;16(3):e03590-24. doi: 10.1128/mbio.03590-24 (PMC11898558; doi:10.1128/mbio.03590-24)

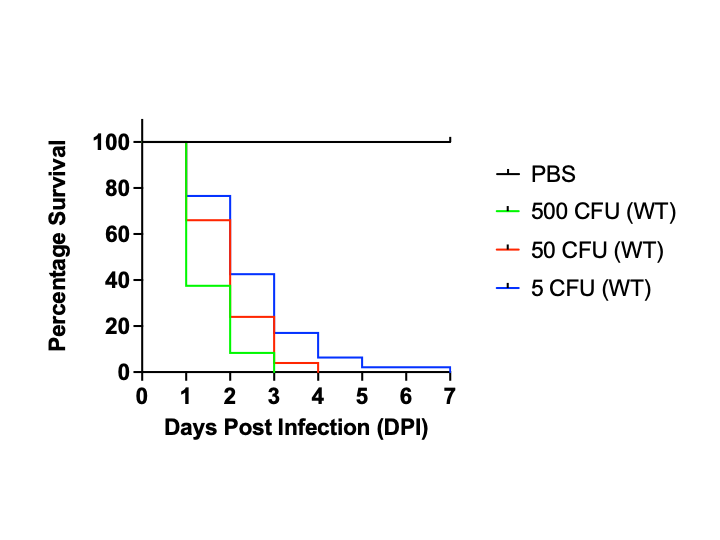

Supplement: Fig S1 — Optimal infectious dose of STm for the CAM tumor model. [file mbio.03590-24-s0002.tiff]

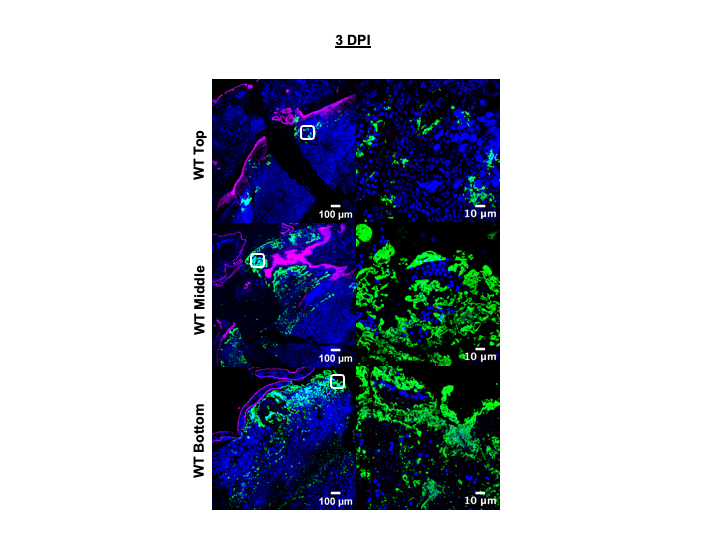

Supplement: Fig S2 — CAM tumor sections infected with WT at 3DPI. [file mbio.03590-24-s0003.tiff]

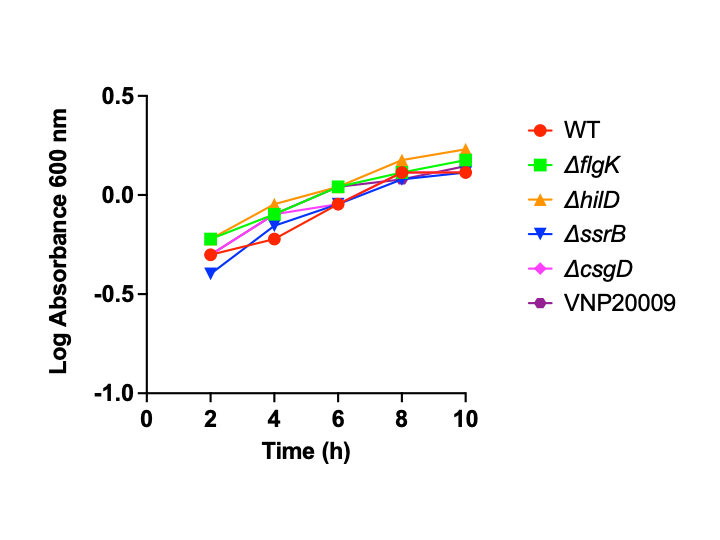

Supplement: Fig S3 — Growth curve of all bacterial strains. [file mbio.03590-24-s0004.tiff]

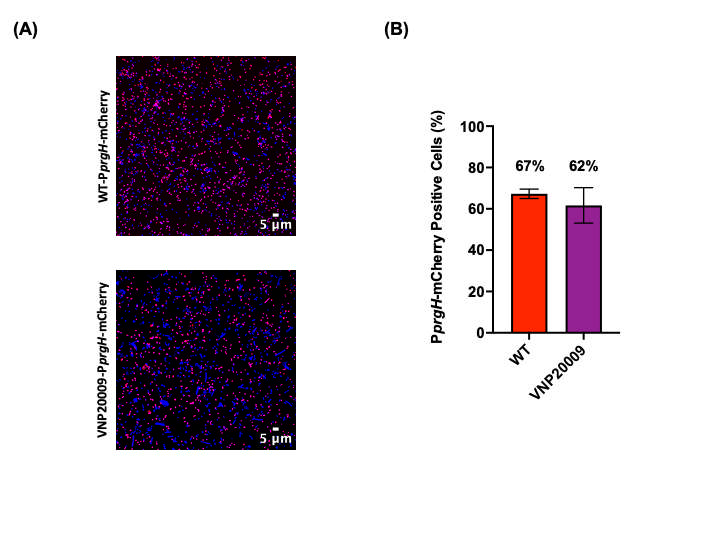

Supplement: Fig S4 — SPI-1 expression in STm WT and VNP20009 strains. [file mbio.03590-24-s0005.tiff]

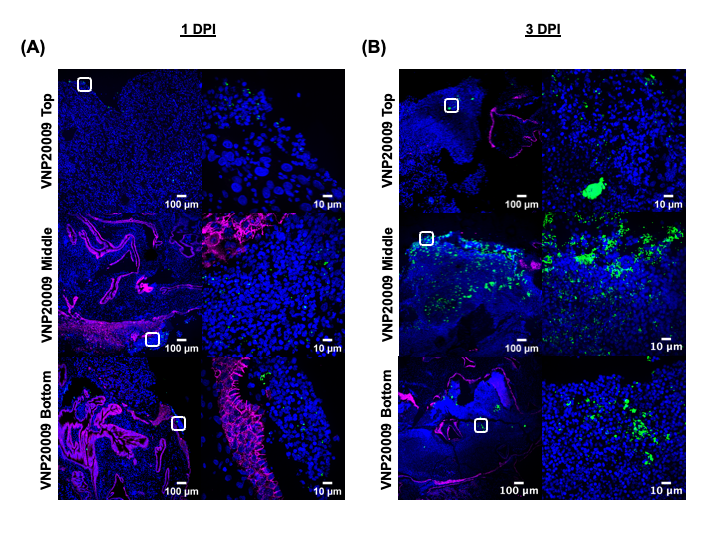

Supplement: Fig S5 — CAM tumor sections infected with VNP20009 at 1- and 3-DPI. [file mbio.03590-24-s0006.tiff]
